# RUNX2 interacts with BRG1 to target CD44 for promoting invasion and migration of colorectal cancer cells

**DOI:** 10.1186/s12935-020-01544-w

**Published:** 2020-10-15

**Authors:** Xiaodong Yan, Dali Han, Zhiqiang Chen, Chao Han, Wei Dong, Li Han, Lei Zou, Jianbo Zhang, Yan Liu, Jie Chai

**Affiliations:** 1 Department of Physiology and Pathophysiology, School of Basic Medical Sciences, Capital Medical University, Beijing, 100069 China; 2 Department of Radiation Oncology, Shandong Cancer Hospital and Institute, Shandong First Medical University and Shandong Academy of Medical Sciences, Jinan, 250117 Shandong Province China; 3 Department of General Surgery, Xuanwu Hospital, Capital Medical University, Beijing, 100069 China; 4 Department of Gastrointestinal Surgery, Heping Hospital Affiliated to Changzhi Medical College, Changzhi, 046000 Shanxi Province China; 5 Internal Medicine-Oncology, Shandong Cancer Hospital and Institute, Shandong First Medical University and Shandong Academy of Medical Sciences, Jinan, 250117 Shandong Province China; 6 Department of Gastrointestinal Surgery, Shandong Cancer Hospital and Institute, Shandong First Medical University and Shandong Academy of Medical Sciences, Jinan, 250117 Shandong Province China; 7 Department of Pathology, Shandong Cancer Hospital and Institute, Shandong First Medical University and Shandong Academy of Medical Sciences, Jinan, 250117 Shandong Province China; 8 Shandong Academy of Occupational Health and Occupational Medicine, Shandong First Medical University and Shandong Academy of Medical Sciences, Jinan, 250117 Shandong Province China; 9 Department of Gastrointestinal Surgery, Shandong Cancer Hospital and Institute, Shandong First Medical University and Shandong Academy of Medical Sciences, No. 440 Ji-Yan Road, Jinan, 250117 Shandong Province China; 10 Tianjin Medical University, Tianjin, 300070 China

**Keywords:** RUNX2, BRG1, CD44, Cancer stem cell, Colorectal cancer

## Abstract

**Background:**

Cancer stem cells (CSCs) play an important role in tumor invasion and metastasis. CD44 is the most commonly used marker of CSCs, with the potential to act as a determinant against the invasion and migration of CSCs and as the key factor in epithelial–mesenchymal transition (EMT)-like changes that occur in colorectal cancer (CRC). Runt-related transcription factor-2 (RUNX2) is a mesenchymal stem marker for cancer that is involved in stem cell biology and tumorigenesis. However, whether RUNX2 is involved in CSC and in inducing EMT-like changes in CRC remains uncertain, warranting further investigation.

**Methods:**

We evaluated the role of RUNX2 in the invasion and migration of CRC cells as a promoter of CD44-induced stem cell- and EMT-like modifications. For this purpose, western blotting was employed to analyze the expression of differential proteins in CRC cells. We conducted sphere formation, wound healing, and transwell assays to investigate the biological functions of RUNX2 in CRC cells. Cellular immunofluorescence and coimmunoprecipitation (co-IP) assays were performed to study the relationship between RUNX2 and BRG1. Real-time quantitative PCR (RT-qPCR) and immunohistochemistry (IHC) were performed to analyze the expressions of RUNX2, BRG1, and CD44 in the CRC tissues.

**Results:**

We found that RUNX2 could markedly induce the CRC cell sphere-forming ability and EMT. Interestingly, the RUNX2-mediated EMT in CRC cell may be associated with the activation of CD44. Furthermore, RUNX2 was found to interact with BRG1 to promote the recruitment of RUNX2 to the CD44 promoter.

**Conclusions:**

Our cumulative findings suggest that RUNX2 and BRG1 can form a compact complex to regulate the transcription and expression of CD44, which has possible involvement in the invasion and migration of CRC cells.

## Background

Cancer stem cell (CSC) is a group of tumor cells that possesses the ability for self-renewal as well as to differentiate among different phenotypes. CSC shares similar characteristics with stem cells, and has been attributed as the root cause of tumor recurrence, metastasis, and treatment tolerance [1]. Accumulating evidences suggest that the growth and metastasis of primary tumors are induced by CSCs [2, 3]. CD44 is one of the most commonly used marker of CSCs that functions essentially as a cell–cell adhesion protein with a key role in the invasion and metastasis of cancer [4].

Tumor metastasis requires a series of interactions between cells and extracellular matrix (ECM) or among cells. As a transmembrane hyaluronic acid receptor and the most commonly used marker of CSCs, CD44 is recognized as a potential determinant against the ability of invasion and metastasis in cancer [5]. As a result, it has received much attention in gastrointestinal tumor research [4, 6, 7]. The epithelial–mesenchymal transition (EMT) is a crucial biological process for the epithelial cancer cells to acquire the ability of migration and invasion [8]. In general, EMT is incomplete and reversible; hence, referring to this transition process as “EMT-like changes” is more appropriate [9]. For most gastrointestinal tumors, EMT-like changes have been reported [10, 11] to decrease the expression of epithelial markers such as E-cadherin, and increase the expression of mesenchymal markers such as N-cadherin [12]. This change in the expression level is occasionally accompanied with an increase in the CD44 expression [13]. CD44-overexpressing cells have been reported to increase EMT-like changes in vitro and decrease the E-cadherin expression, while inducing the expression of EMT markers in colon cancer cells [13].

The process of occurrence and development of colorectal cancer (CRC) involves multiple genes. Cells with increased oncogenic functions and the loss of tumor-suppressor gene functions demonstrate uncontrolled inhibition of proliferation, differentiation, and apoptosis [14, 15]. Therefore, we aimed to determine more highly specific CRC-related genes in clinical practice so as to provide the basis for the diagnosis, targeted treatment, and prognosis risk assessment of CRC through the combined detection of multiple genes. It is presently believed that the abnormal expression of RUNX2 is involved in the CRC progression [16]. RUNX2 is one of the important members of the RUNX transcription factor family, and involved in the regulation of cell proliferation and differentiation [17]. RUNX2 is highly expressed in pancreatic cancer, liver cancer, and other malignant tumor tissues; hence, the knockdown of RUNX2 expression can inhibit its malignant biological behavior in cell experiments [18, 19]. As a mesenchymal stem marker for cancer [20], RUNX2 is involved in stem cell biology and tumorigenesis [21], and, possibly, in the regulation of CSC [22]. Moreover, it has been suggested that RUNX2 can promote the invasion and metastasis of non-small cell lung cancer through EMT-related pathways [23]. However, whether RUNX2 is involved in CSC and in producing EMT-like changes in CRC remains uncertain, warranting further investigation.

In this study, we comprehensively analyzed the possible mechanism of RUNX2 in the invasion and migration of CRC cells and attempted to ascertain whether RUNX2 monitors the sphere-forming ability and EMT of CRC cells by targeting CD44. In addition, RUNX2 can interact with *brahma-related gene 1* (BRG1)—a key regulator of CD44 and a major transcriptional regulator [24, 25]—to promote the invasion and migration processes via the regulation of CD44 in CRC cells. The outcomes of clinical cases and analysis of the cBioPortal for Cancer Genomics database also demonstrated the significant positive correlation among RUNX2, BRG1, and CD44 expressions in colon cancer tissues. Further understanding of the role of RUNX2 in tumor development is expected to promote the progress of strategies of multigene combined diagnosis and treatment for CRC.

## Materials and methods

### Human colorectal specimens

The CRC and adjacent tissues were obtained from the Shandong Cancer Hospital and Institute, Shandong First Medical University and Shandong Academy of Medical Sciences during 2010–2013. All samples were stored in liquid nitrogen at –80 °C immediately after collection.

### Cell culture and transfection

Human colon cancer RKO and HT115 cell lines were sourced from the European Collection of Cell Cultures (ECACC; Salisboury, UK), while HT29, SW620, and SW480 cells were sourced from ATCC (Manassas, VA, USA). HEK293T cells were purchased from the Kunming Cell Bank, Chinese Academy of Sciences (Kunming, China). Dulbecco's Modified Eagle Medium (DMEM; Hyclone, Logan, UT) was used as the cell culture medium for RKO, HT115, and HEK293T, while McCoy's 5a Medium Modified (Gibco, USA) was used for HT-29. Leibovitz's L-15 Medium (Gibco, USA), supplemented with penicillin (100 U/mL; Solarbio, Beijing, China), streptomycin (100 μg/mL; Solarbio, Beijing, China), and heat-inactivated 10% fetal bovine serum (FBS; Gibco, USA) was used as the feed medium for SW620 and SW480. The CRC cells were cultured at 37 °C under the atmosphere of 5% CO_2_ and 95% humidity, with the fusion rate maintained at > 80%. The cells were harvested as described in the next section.

Small-interfering RNA (siRNA) duplexes were transfected to CRC cells up to 30–50% confluency with Lipofectamine 3000 (Invitrogen Life Technologies, USA). siRNA specific for human RUNX2 was obtained from Santa Cruz Biotechnology (sc-37145). RUNX2 siRNA (h) is a pool of 3 different siRNA duplexes, A-sense: CCAUAACCGUCUUCACAAAtt, UUUGUGAAGACGGUUAUGGtt (antisense); B-sense: CCUUCCACUCUCAGUAAGAtt, UCUUACUGAGAGUGGAAGGtt (antisense); C-sense: and CACUCCAUAUCUCUACUAUtt, AUAGUAGAGAUAUGGAGUGtt (antisense). The siRNA-specific sense strands for human BRG1 is given elsewhere [26]: siRNA-1: 5′-GGGUACCCUCAGGACAACATT-3′ and siRNA-2: 5′-CGACGUACGAGUACAUCAUTT-3′. For CD44 knockdown, the sense sequences for CD44 siRNA were prepared as described previously [27]. CD44 siRNA (a pool of two): 5′-CAGAAACTCCAGACCAGTT-3′ and 5′-AATGGTGCATTTGGTGAAC-3′. The BRG1 and CD44 siRNAs were synthesized by the Shanghai Heyuan Company. RUNX2 (NM_001024630) Human-Tagged ORF Clone (CAT #RC212884) and BRG1 (SMARCA4) (NM_001128849) Human-Tagged ORF Clone (CAT #RG226420) were purchased from OriGene (OriGene, USA).

### Sphere formation assay

The sphere-formation assay was performed as described elsewhere [28]. Briefly, after removing the serum-containing medium, the well-grown RKO and HT115 cells were digested, centrifuged, and washed twice with sterile phosphate-buffered saline (PBS) (pH 7.3). These cells were then resuspended in 1 X serum-free B27 supplement DMEM/F-12 medium containing 20 mg/L epidermal growth factor and 20 mg/L basic fibroblast growth factor. The cells were cultured in 6-well ultra-low attachment plate (Corning, Kraemer, CA) at a density of 5000 cells/well and incubated at 37 °C under the atmosphere of 5% CO_2_ for 7–10 days. The tumors spheres were pictured and quantified under a microscope (Olympus; Tokyo, Japan). The number and diameter of the spheres were calculated using the Image Pro Plus version 6.0.

### Western blotting

The cells from each group that were cultured for 48 h after siRNA or plasmid transfection were lysed in RIPA buffer (C1053; Applygen Technologies Inc., Beijing, China) containing a protease inhibitor cocktail (Roche, Switzerland) and phenylmethane-sulfonyl fluoride (PMSF) (Sigma, USA). The protein concentration was determined using a BCA protein assay kit (Thermo Scientific, USA). Protein samples were separated by 8–12% sodium dodecyl sulfate–polyacrylamide gel electrophoresis (SDS-PAGE) depending on the molecular weight of the protein of interest with the SDS-PAGE Running Buffer (B1005; Applygen Technologies Inc., Beijing, China). Proteins were transferred onto polyvinylidene fluoride membranes and incubated at 4 °C overnight with primary antibodies. Thereafter, the membranes were incubated with horseradish peroxidase-containing goat anti-rabbit or goat anti-mouse IgG antibody (Cell Signaling Technology, USA) for 1 h at the room temperature. The proteins were developed with an enhanced chemiluminescence solution (Key GEN Bio TECH, China) through the AI600 Images System (General Electric Company, USA). In addition, β-actin (Cell Signaling Technology, USA) was used as the internal control. The specific antibody information is listed in Additional file 1. Table S1.

### The transwell invasion assay

After transfection for 48 h, the transwell assay was performed in a modified Boyden chamber fitted with 8-μm pore filters. Briefly, CRC cells (5 × 10^4^ cells/well) treated under different conditions were added to the upper chamber, and the cell culture medium was added to the lower chamber, the chambers were then incubated at 37℃ under the atmosphere of 5% CO_2_ and 95% humidity for 24 h. After 24-h incubation, the cells were passed through the Matrigel-coated membrane, stained, and, finally, fixed. The cells from randomly selected fields were counted under the microscope. The assay was repeated thrice.

### Wound healing assay

Cells treated under different conditions were plated at an equal density into 6-well plates and cultured to 80% confluency at 37 °C in a 5% CO_2_ humidified chamber. A few cells were scratched from the plate with gentle scratching using a small sterile pipette tip. The detached cells were then washed twice in PBS solution and supplemented with fresh medium. The new plates were incubated at 37 °C under 5% CO_2_ humidified chamber for 48 h. The degree of cell migration was quantified based on their ability to close the artificially created gaps and record the same under an optical microscope. The change in the scratch area with time and the wound healing percent were calculated based on the initial scratch area, as given below:

Wound healing (%) = The ratio of the area healed at 48 h (the initial scratch area—the scratch area after 48 h)/the initial scratch area.

### Cellular immunofluorescence

RKO and HT115 cells were seeded onto coverslips placed into a 6-well plate at a concentration of 2 × 10^4^/mL, followed by incubation at 37 °C under 5% CO_2_ humidified atmosphere for 48 h and then washing the adherent cells with sterile PBS thrice. Next, the PBS was discarded and 4% paraformaldehyde was added to each well for 20 min at the room temperature. The cells were washed three more times with sterile PBS, and the slides were blocked with methanol containing 3% hydrogen peroxide for 10 min. The samples were then rinsed thrice with PBS, permeabilized with 0.25% Triton X-100 (9002931; AMRESCO, USA) in PBS for 10 min, and blocked with 5% normal goat serum (ZLI-9056; ZSGB-bio) in PBS for 60 min. After blocking, the cells were incubated with the corresponding primary antibodies RUNX2 (ab76956; Abcam, UK) and BRG1 (21634–1-AP; Proteintech, USA) overnight at 4 °C, followed by treatment with anti-mouse-Alexa Fluor 594 and anti-rabbit-Alexa Fluor 488 secondary antibody, respectively, for 1 h at the room temperature. Next, 4′,6-diamidinophenyl-indole (DAPI) was used to stain the cell nuclei for 5 min, and the slides were observed under a confocal microscope (TCS-SP5; Leica, Mannheim, Germany). The specific antibody information is provided in Additional file 1. Table S1.

### Coimmunoprecipitation (co-IP) assay

The CRC RKO cells or transfected HEK293T cells were harvested and lysed in IP lysis buffer (C1054; Applygen Technologies Inc.) with PMSF (ST506; Beyotime Biotechnology, Shanghai, China) and protease inhibitor cocktail (Roche, Switzerland) for 30 min on an ice bath. Next, 100μL of the sample was used as the Input, and the other sample for the subsequent co-IP. The sample was centrifuged at 14,000 rpm at 4 °C for 15 min to remove the insoluble materials.

The supernatants were incubated with 1 μg of anti-RUNX2 or anti-BRG1 antibodies/mg protein for the extracted cells and with 1 μg of anti-Myc or anti-GFP antibodies/mg protein for protein extraction from the transfected HEK293T cells with a gentle rotation on the rotary table at 4 °C for 8–12 h. Then, the supernatants were collected on Protein A/G immunoprecipitation beads at 4 °C for 3–5 h, followed by extensive washing on the rotary table. IP with rabbit (3900S, Cell signaling Technology, USA) or mouse (sc-2025, Santa Cruz Biotechnology, USA) IgG (IgG-IP) was used as the negative control. The specific antibody information is provided in Additional file 1. Table S1.

### Chromatin immunoprecipitation (CHIP) assay

The CHIP Assay Kit (56383; Cell Signaling Technology, USA) was used to perform the CHIP assay as instructed by the manufacturer. Rabbit polyclonal anti-RUNX2 antibodies (12556S; Cell Signaling Technology, USA) or rabbit anti-IgG antibodies (3900S; Cell Signaling Technology, USA) was used for precipitation, and the IP was purified on protein A/G immunoprecipitation beads. The DNA purified from CHIP was ligated with the adapter and subjected to PCR amplification as per the manufacturer's instructions (Illumina; San Diego, CA, USA). We used the ConSite service based on the JASPAR datasets for this purpose. Transcription binding site prediction of RUNX2 to the CD44 promoter was performed as described elsewhere [29]. The primer set used to amplify the promoter regions of CD44 was: 5′-GGACAGATGGGAAATGAGTGGA-3′ (sense) and 5′-TTCATCCAACCACACACCTTTT-3′ (antisense). The specific antibody information is provided in Additional file 1. Table S1.

### Real-time quantitative PCR (RT-qPCR)

The CRC tissues were extracted with the TRIzol reagent (Invitrogen Life Technologies; Chicago, USA). RT-qPCR was performed with specific primers (Qiagen; Hilden, Germany) and the SYBR-Green PCR Master Mix Kit (Takara, Japan) on the ABI 7500 System (Applied Biosystems; Foster City, CA, USA). The primers of CD44 and BRG1 were used as suggested in previous studies [24, 30]. CAPDH was used for gene expression normalization. All primers were sourced from the Sangong Biotech (Shanghai, China). The primers used in this study are listed in Additional file 1. Table S2.

### Immunohistochemistry (IHC)

The expression patterns of RUNX2, BRG1, and CD44 in the CRC tissues and paracancerous tissues were assessed by the IHC method, as reported by Wu et al. [31]. IHC staining for RUNX2 (ab76956; Abcam, UK), SMARCA4/BRG1 (21634–1-AP; Proteintech, USA), and CD44 (BBA10; R&D Systems, USA) was performed on the CRC paraffin sections. Dewaxed tissue arrays and antigens were retrieved under high pressure. Blocking of endogenous peroxidases was performed with 3% hydrogen peroxide for 10 min at the room temperature. The slides were then washed twice with PBS and then blocked with 5% BSA for 20 min at the room temperature. The tissues were then incubated with the primary antibody at 4 °C overnight, followed by washing thrice with PBS. The secondary antibody was incubated at 37 °C for 30 min, followed by washing thrice with PBS. Then, the sections were incubated with horseradish peroxidase complex for 30 min at 37 °C and visualized with diaminobenzidine (DAB). All immunohistochemical images were captured by the Olympus B × 51 microscope (Olympus, Tokyo, Japan) and the DP 50 Camera (Olympus). Images were processed using the DPC Controller Software (Olympus). In the tissue section, all dark brown stained cells were strongly positive, brown-yellow stained cells were moderately positive, light-yellow stained cells were weakly positive, and blue stained cells were negative. Then, the areas of strong-positive, moderate-positive, weak-positive, and negative (unit: pixel), the percent of positive cells, and the final histochemistry score (H-score) were obtained via the histological scoring method for analyzing the immunohistochemistry data. The number of positive cells and their staining intensity in each section were transformed into the corresponding values for semi-quantitative staining, as given below: H-score = ∑ (PI × I) = (percent of cells of peak intensity × 1) + (percent of cells of model intensity × 2) + percent of cells of strong intensity × 3). Where PI percent of positive cells among total cells in a particular section and I = staining intensity. The specific antibody information is provided in Additional file 1. Table S1.

### Statistical analysis

Statistical analyses were performed using the GraphPad Prism 5.01 Software (GraphPad Software, La Jolla, CA). Data were expressed as the mean ± SE and were analyzed using Student’s t-tests. *P* < 0.05 indicated statistically significant values.

## Results

### RUNX2 contributes to stem-like properties of CRC cells

In CRC, the expression of RUNX2 is upregulated and closely associated with the clinical stages and liver metastases [16]. In addition, RUNX2 knockout could significantly reduce the capabilities of CRC proliferation, migration, and invasion in SW480 and DLD-1 cells [16]. Based on the present knowledge, CSCs play a key role in the initiation and growth of cancer. They also play an important role in determining the formation, recurrence, and metastasis of cancer. CD44 is a commonly used and extensively studied marker of CSCs in CRC [5, 13].

**Fig. 1:**
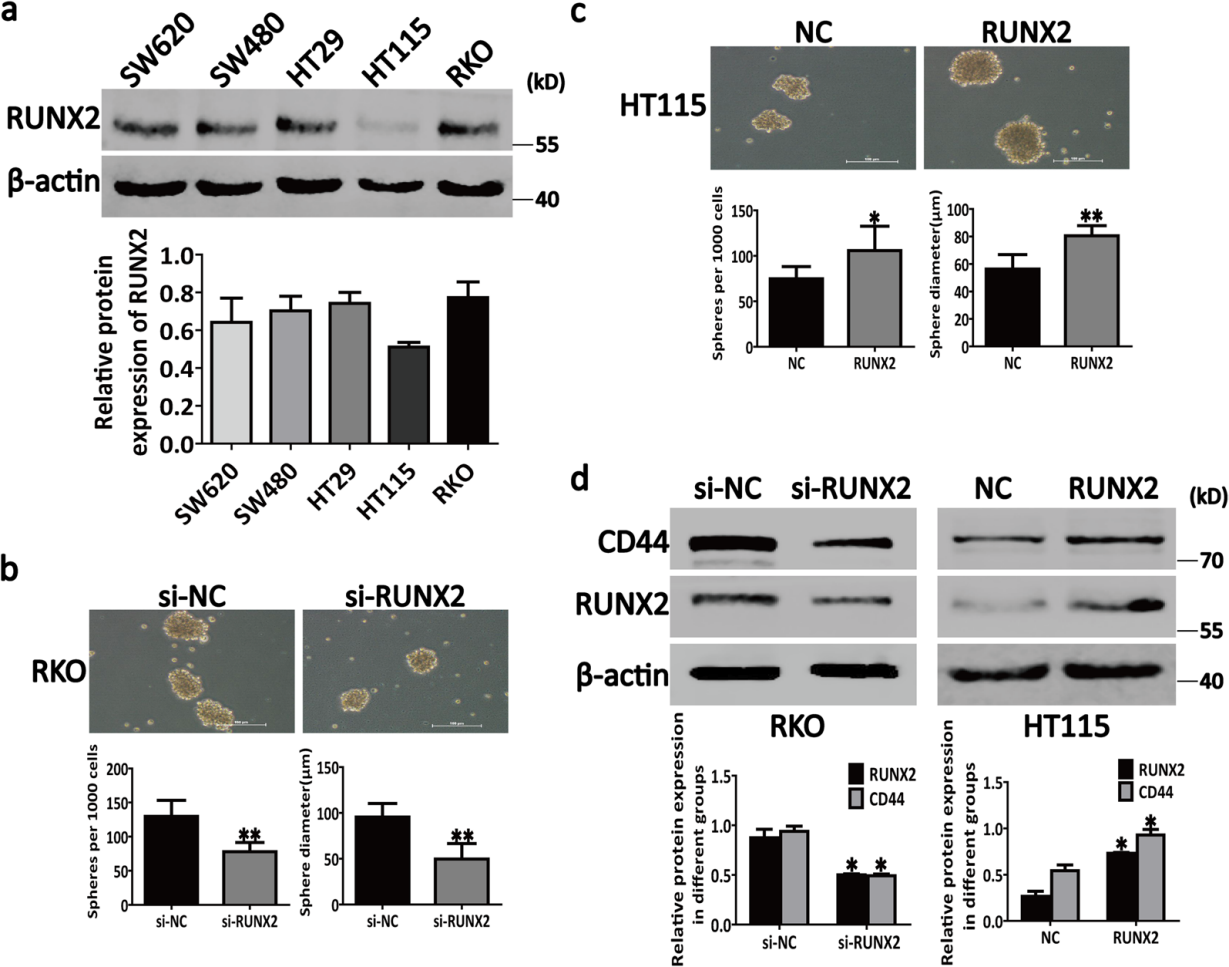
RUNX2 possibly monitors the sphere-forming ability of CRC cells by targeting CD44. **a** Endogenous expression of RUNX2 in different CRC cells. **b** The analysis of sphere-forming ability by sphere formation assays in RKO cells under different conditions. **c** The analysis of sphere-forming ability by sphere formation assays in HT115 cells under different conditions. **d** Western blotting results showing that RUNX2 can regulate the CD44 expression. Data are shown as means ± SE; *P<0.05, **P<0.01; *NC* negative control, *si* short interfering, *RUNX2* overexpression of RUNX2

Whether RUNX2 participates in the regulation of CSC characteristics in CRC remains unclear. To explore this point, we first tested the protein expression of RUNX2 in several CRC cell lines (Fig. 1a). Our data revealed that the lowest expression of RUNX2 was in the HT115 cells among the 5 CRC cell lines and that the RUNX2 expression in RKO cells was higher than that in other cell lines (Fig. 1a). Therefore, we selected RKO and HT115 cells for the further studies.

Next, we transfected RUNX2 si-RNA and RUNX2 overexpression plasmids into RKO and HT115 cells, respectively, in order to inhibit and overexpress RUNX2. Our results suggested that the knockdown of RUNX2 could markedly inhibit the sphere-forming ability of RKO cells (Fig. 1b) and cause a marked downregulation of CD44 in RKO cells (Fig. 1d). On the contrary, the overexpression of RUNX2 could markedly enhance the sphere-forming ability of HT115 cells (Fig. 1c) and increase the expression of CD44 in HT115 cells (Fig. 1d). These results indicate that RUNX2 possibly monitors the sphere-forming ability of CRC cells by targeting CD44.

### RUNX2 promotes CD44-induced CRC cell EMT

**Fig. 2:**
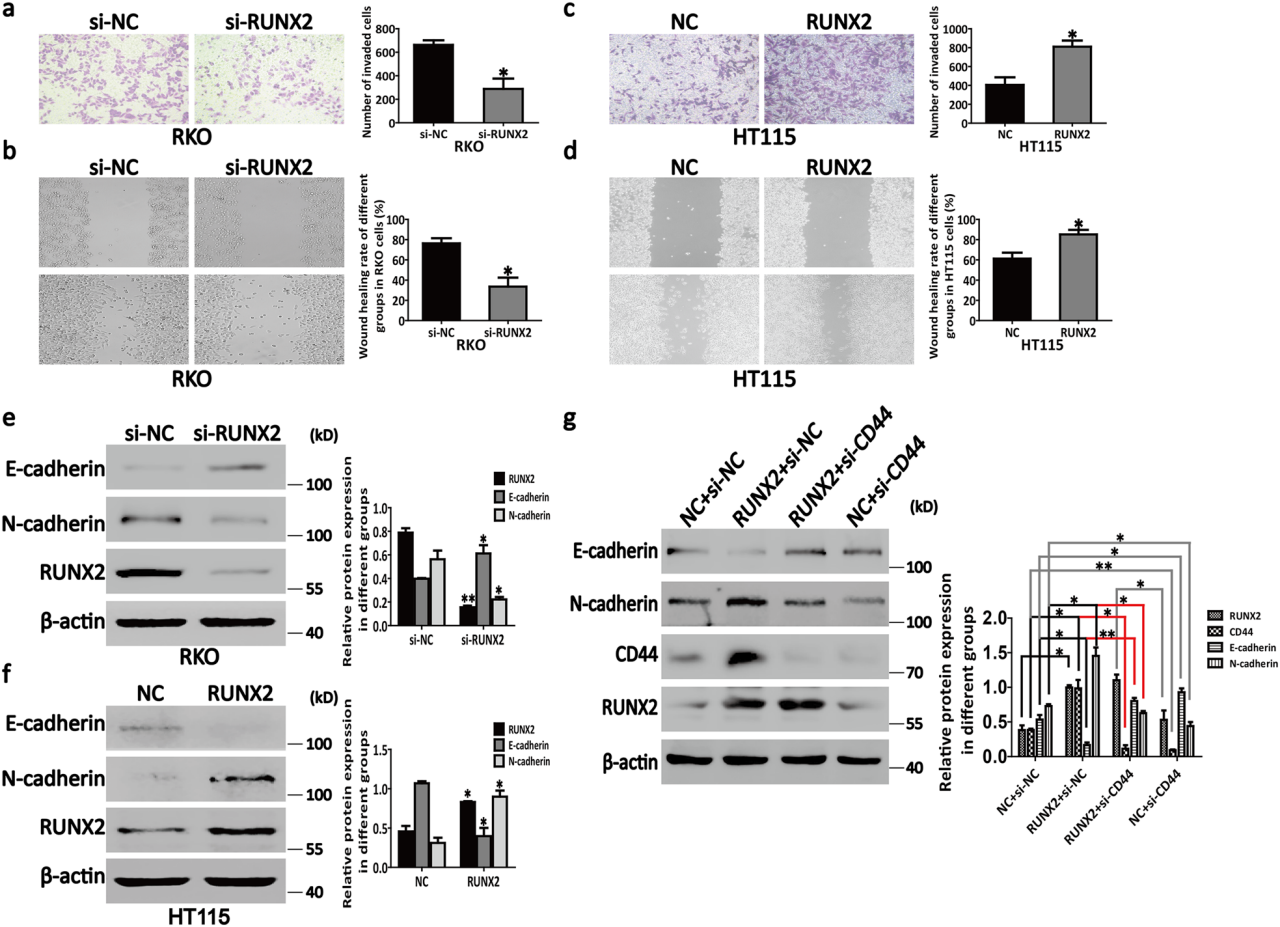
RUNX2 promotes CD44-induced EMT in CRC cells. **a**, **c** The number of invading cells was determined by using the transwell assay in RKO and HT115 cells. **b**, **d** The wound healing rate of RKO and HT115 cells was measured by the wound healing assay. **e** Knockdown of RUNX2 promoted E-cadherin expression and inhibited N-cadherin expression in RKO cells. **f** Overexpression of RUNX2 inhibited E-cadherin expression and promoted N-cadherin expression in HT115 cells. **g** Knockdown of CD44 increased E-cadherin expression and decreased N-cadherin expression in RUNX2-overexpressed HT115 cells. Data are shown as means ± SE; *P<0.05, **P<0.01; *NC* negative control, *si* short interfering, *RUNX2* overexpression of RUNX2

RUNX2 affects the biological behavior of cells by regulating several downstream target genes’ expressions. In the recent years, several researches have indicated that changes in the RUNX2 expression are closely associated with the occurrence and development of malignant tumors [32–34]. The EMT is the key to tumor invasion and metastasis [35]. However, it remains unclear whether RUNX2 contributes to EMT of CRC cells. To explore this point, we ran experiments wherein RUNX2 was inhibited and overexpressed in RKO and HT115 cells separately. As shown in Fig. 2a, b, the results of transwell and wound healing assays suggested that RUNX2 knockdown in RKO could sufficiently abate the cells invasiveness and migratory ability, and vice versa in HT115 cells (Fig. 2c, d). In addition, the inhibition of RUNX2 could increase the E-cadherin expression and decrease the N-cadherin expression in these cells relative to those in untreated cells; this observation is consistent with that for EMT progression (Fig. 2e). Moreover, an opposite result was observed after the overexpression of RUNX2 in HT115 cells (Fig. 2f). These cumulative results suggest that RUNX2 could promote EMT in CRC cells.

Furthermore, as a transmembrane glycoprotein, CD44 plays the key role in CRC cell adhesion, migration, and metastasis [4] as well as in the EMT-like modifications in colon cancer cells [36]. These facts support that RUNX2 probably interacts with CD44, suggesting that CD44 mediates the RUNX2 regulation of EMT in CRC cells. To confirm whether RUNX2 can promote EMT in CRC cells via the CD44 pathway, we used siRNA to knock down CD44 while overexpressing RUNX2 in HT115 cells. Western blotting confirmed that the expression levels of E-cadherin increased with CD44 knockdown (Fig. 2g). As a result, the ability of RUNX2 to promote N-cadherin upregulation in these cells also markedly reduced with the CD44 knockdown (Fig. 2g).

### RUNX2 interacts with BRG1 to promote CD44 transcription

**Fig. 3:**
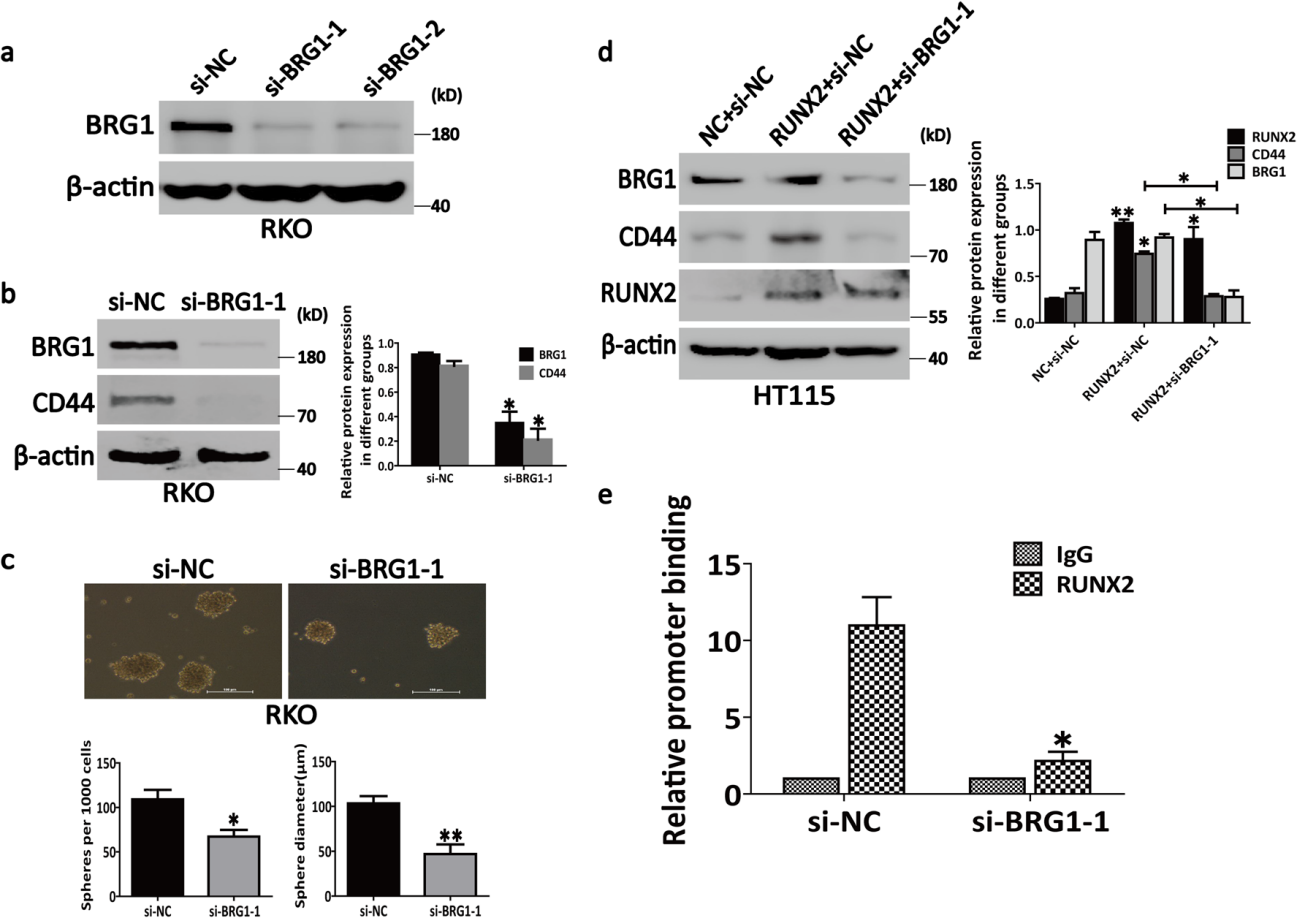
RUNX2 interacts with BRG1 to promote CD44 transcription. **a** Both the siRNAs for BRG1 could effectively inhibit the expression of BRG1. **b** BRG1 knockdown decreased the expression of CD44. **c** BRG1 knockdown inhibited the sphere-forming ability of RKO cells. **d** Knockdown of BRG1 offset the ability of RUNX2 to promote the expression of CD44 in HT115 cells. **e** The analysis of RUNX2 binding to the CD44 promoter by CHIP assay in RKO cells. Genomic DNA was immunoprecipitated with anti-RUNX2 antibody, where IgG served as a negative control. The primer set specific to the RUNX2 binding site of CD44 promoter was used in real-time PCR amplification. Data are shown as means ± SE; *P<0.05, **P<0.01; *NC* negative control, *si* short interfering, *RUNX2* overexpression of RUNX2

As the key regulator of CD44, BRG1 is known to promote the expression of CD44 and play a crucial role in the invasion and migration of cancer cells [24, 37]. Consistent with this fact, our results showed that the knockdown of BRG1 with si-BRG1 (Fig. 3a) could decrease the CD44 expression (Fig. 3b) and inhibit the sphere-forming ability of RKO cells (Fig. 3c), which in turn indicated the necessity of BRG1 for the transcription and expression of CD44. In addition, as a component of the SWI/SNF chromatin-remodeling complex, BRG1 is involved in the transcriptional activity and is, in fact, a major regulator of transcription [38].

A previous study reported that BRG1 is markedly increased in CRC, thereby promoting the occurrence and development of CRC [39]. However, whether BRG1 interacts with the transcription factor RUNX2 to increase the transcriptional activity of CD44 to promote the invasion and migration of CRC cells remains unclarified. To clarify this point, we knocked down BRG1 while overexpressing RUNX2 in HT115 cells. Western blotting revealed that the knockdown of BRG1 offset the ability of RUNX2 to promote the expression of CD44 (Fig. 3d). Furthermore, in CHIP assays, we first found that RUNX2 could bind to the fragment of CD44 promoter, which contained the binding sites of RUNX2 (Fig. 3e; left panel). The knockdown of BRG1 could block the binding of RUNX2 to the CD44 promoter (Fig. 3e; right panel). In summary, the cumulative observations suggest that BRG1 contributes to the recruitment of RUNX2 to the CD44 promoter.

### RUNX2 coimmunoprecipitates with BRG1 from CRC cells and an expression system

**Fig. 4:**
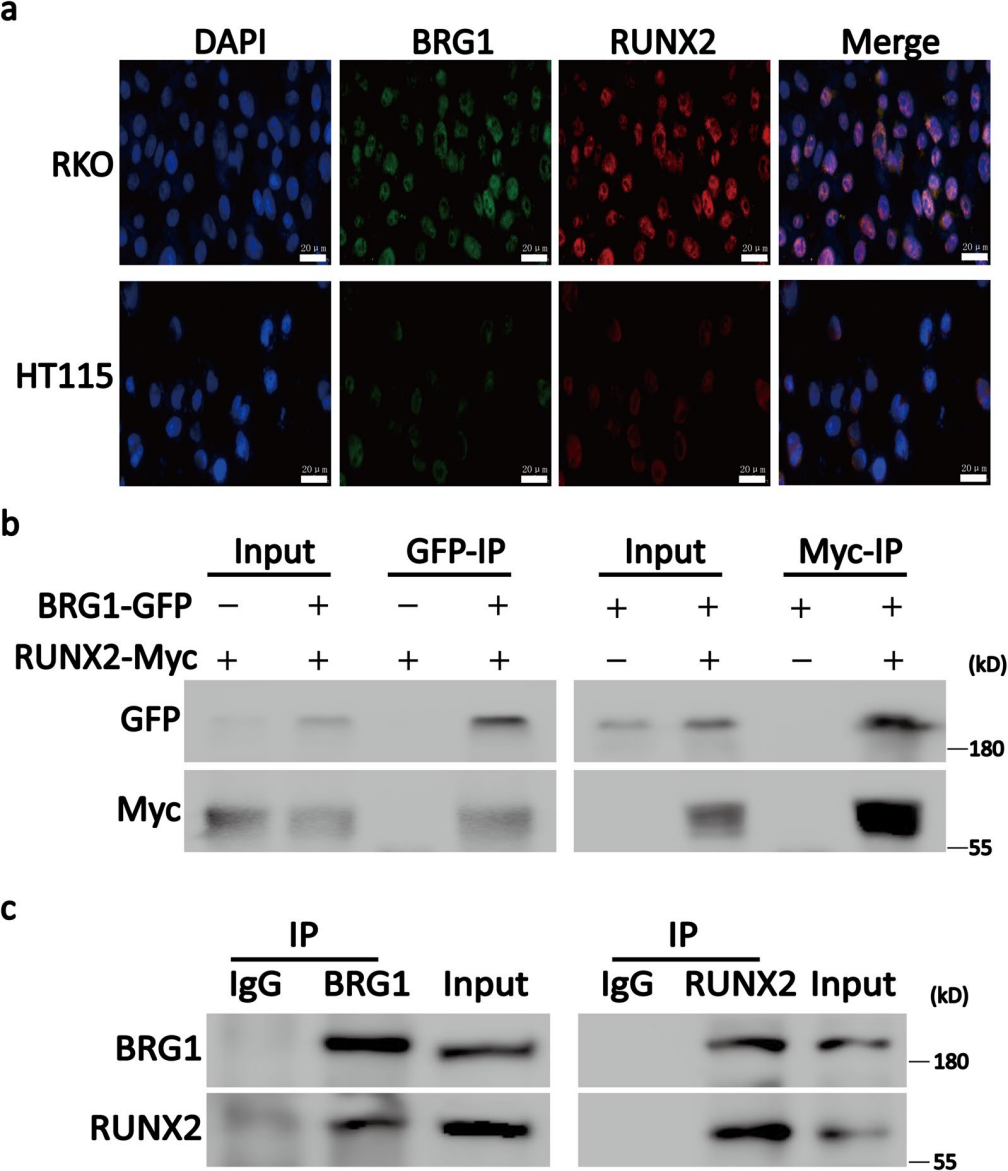
RUNX2 interacts with BRG1. **a** Confocal microscopy results showing that both RUNX2 and BRG1 were expressed in the nucleus of RKO and HT115 cells. **b** Co-IP experiments were performed with cell lysates of HEK293T expressing RUNX2-Myc and/or BRG1-GFP. The transfected constructs are indicated in the top panel. Anti-GFP (GFP-IP; left panel) or anti-Myc (Myc-IP; right panel) antibody was used for the immunoprecipitation assay. The antibodies used for WB are described on the left panel. **c** Co-IP experiments were performed on RKO cell lysates. The immunoprecipitation assay of RKO cell lysates with RUNX2, BRG1, or control (IgG) antibody. RUNX2 or BRG1 was detected by WB using the indicated antibody

Next, we aimed to investigate whether RUNX2 and BRG1 interact physically. For this purpose, we first analyzed the expression and localization of RUNX2 and BRG1 in RKO and HT115 cells through confocal microscopy. The confocal microscopy revealed both the proteins were expressed in the nucleus of CRC cells, thereby indicating the position condition of their interaction (Fig. 4a).

In order to clarify the interaction between RUNX2 and BRG1, exogenous co-IP experiments were conducted in RUNX2-Myc-DDK- and/or BRG1-GFP-overexpressing HEK293T cells (Fig. 4b). This experiment confirmed the RUNX2-BRG1 interaction by immunoprecipitation assays in HEK293T cells with exogenously expressing tagged proteins. To clarify whether the interaction between RUNX2 and BRG1 also occur in CRC cells, we used RKO cells to perform endogenous co-IP experiments. Consistent with the exogenous results, the endogenous test also demonstrated that RUNX2 and BRG1 form a compact complex (Fig. 4c). Together, these results demonstrated that RUNX2 and BRG1 can form a compact complex in CRC cells.

### RUNX2 positively correlates with BRG1 and CD44 in CRC

**Fig. 5:**
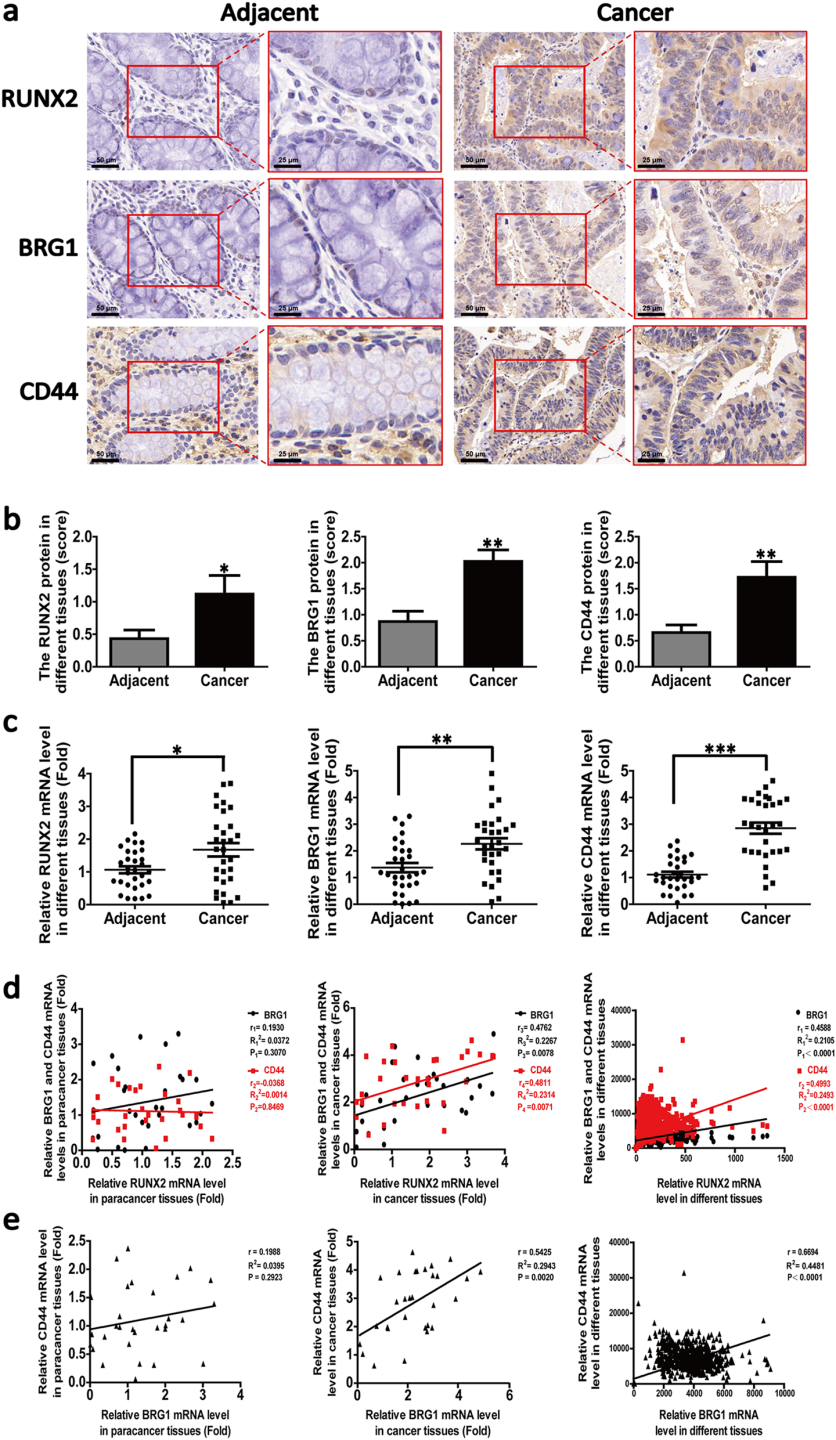
RUNX2 positively correlates with BRG1 and CD44 in cancer tissues. **a** Representative images of IHC staining for RUNX2, BRG1, and CD44 in human CRC tissue samples (magnification, 200 × , 400 ×). **b** Semi-quantitative results of RUNX2, BRG1, and CD44 expression levels in CRC tissues (n = 9 pairs). **c** The expression of RUNX2, BRG1, and CD44 in different tissues by RT-PCR (n = 30 pairs). **d** The correlation among RUNX2 and BRG1, CD44 in different colorectal tissues (n = 30 pairs), and the analysis of correlation among them based on the CRC data of cBioPortal for Cancer Genomics database (n = 942). **e** The correlation between BRG1 and CD44 in different colorectal tissues (n = 30 pairs) and the analysis of correlation between them in the CRC data of cBioPortal for Cancer Genomics database (n = 942). Data are shown as means ± SE. *P<0.05, **P<0.01, ***P<0.001

Next, we attempted to investigate the relationship among RUNX2, BRG1, and CD44 in human CRC tissues. For this purpose, we first evaluated the levels of RUNX2, BRG1, and CD44 in the CRC tissues in comparison with those in the paracancerous tissues. We found that both of them were markedly increased (Fig. 5a, b) by using IHC assay (n = 9 pairs). To verify this result, we detected the gene expressions of RUNX2, BRG1 and CD44 in CRC and paracancerous tissues by RT-qPCR. The expression levels of RUNX2, BRG1, and CD44 were markedly increased in the CRC tissues in comparison with those in the paracancerous tissues (Fig. 5c; n = 30 pairs).

Moreover, we analyzed the correlation among the mRNA expression of RUNX2, BRG1, and CD44. We noted no significant correlation among them in the paracancerous groups (Fig. 5d; left panel). However, a significant positive correlation was noted among them in the cancerous groups (Fig. 5d; middle panel). To validate this finding, we used the corresponding RNA-Seq data to analyze the correlation among RUNX2, BRG1, and CD44. The analysis of 942 cases of cancer tissues revealed a significant positive correlation among RUNX2, BRG1, and CD44 (Fig. 5d; right panel). Similarly, we analyzed the correlation between BRG1 and CD44 found a significant positive correlation between the expression levels of BRG1 and CD44 in the cancerous groups (Fig. 5e). Corresponding RNA-Seq data were obtained from the cBioPortal for Cancer Genomics database (https://www.cbioportal.org/) [40].

## Discussion

Tumor recurrence and metastasis are major causes of death for cancer patients. The 5-year survival rate of advanced CRC patients with distant metastasis is extremely low [41]. The key to the prevention and treatment of recurrence and metastasis of CRC is to explore the mechanisms of tumor invasion and metastasis. Accumulating evidences have confirmed the oncogenic role of RUNX2 in several cancers, and it has also been associated with the prognosis of patients with recurrence and metastasis [42–46]. Therefore, we investigated the potential role of RUNX2 in human CRC. Interestingly, we identified a new function of RUNX2 in regulating CSCs, as well as revealed a novel mechanism of RUNX2 in monitoring cell invasion and migration in CRC cells. Thus, CSCs are the key factors involved in tumor recurrence and metastasis. Our results demonstrated that the knockdown of RUNX2 inhibited the expression of CD44 and markedly reduced the sphere-forming ability in RKO cells. In contrast, the overexpression of RUNX2 exerted the opposite effects in HT115 cells. These findings together provide novel evidence supporting the contribution of RUNX2 to the regulation of CSCs in CRC.

EMT is an important biological process involved in the maintenance of the stemness of CSCs as well as in the invasion and metastasis. The typical biological changes in EMT are the decreased expression of epithelial marker E-cadherin and the increased expression of mesenchymal marker N-cadherin [35].

The transwell and wound healing experiments conducted in this study suggested that the knockdown of RUNX2 was sufficient to reduce the invasiveness and the migratory ability of RKO cells (Fig. 2a, b) and vice versa in HT115 cells (Fig. 2c, d). Meanwhile, the knockdown of RUNX2 promoted the upregulation of E-cadherin (epithelial marker) and the downregulation of N-cadherin (mesenchymal marker) in RKO cells and vice versa in HT115 cells (Fig. 2e, f). In addition, as the major marker of CSCs, CD44 is involved in the EMT regulation of CRC cells [36]. To further prove that CD44 plays an important role in the regulation of RUNX2-mediated EMT in CRC cells, we knocked down CD44 while overexpressing RUNX2 in HT115 cells. The knockdown of CD44 while overexpressing RUNX2 promoted the E-cadherin expression but inhibited the N-cadherin expression, while a simple overexpression of RUNX2 in HT115 cells was associated with CD44-dependent EMT-like changes (Fig. 2g). Together, our observations and findings indicate that RUNX2 can promote the EMT through the CD44 pathway, suggesting that this is the key mechanism by which RUNX2 promotes CRC progression.

**Fig. 6:**
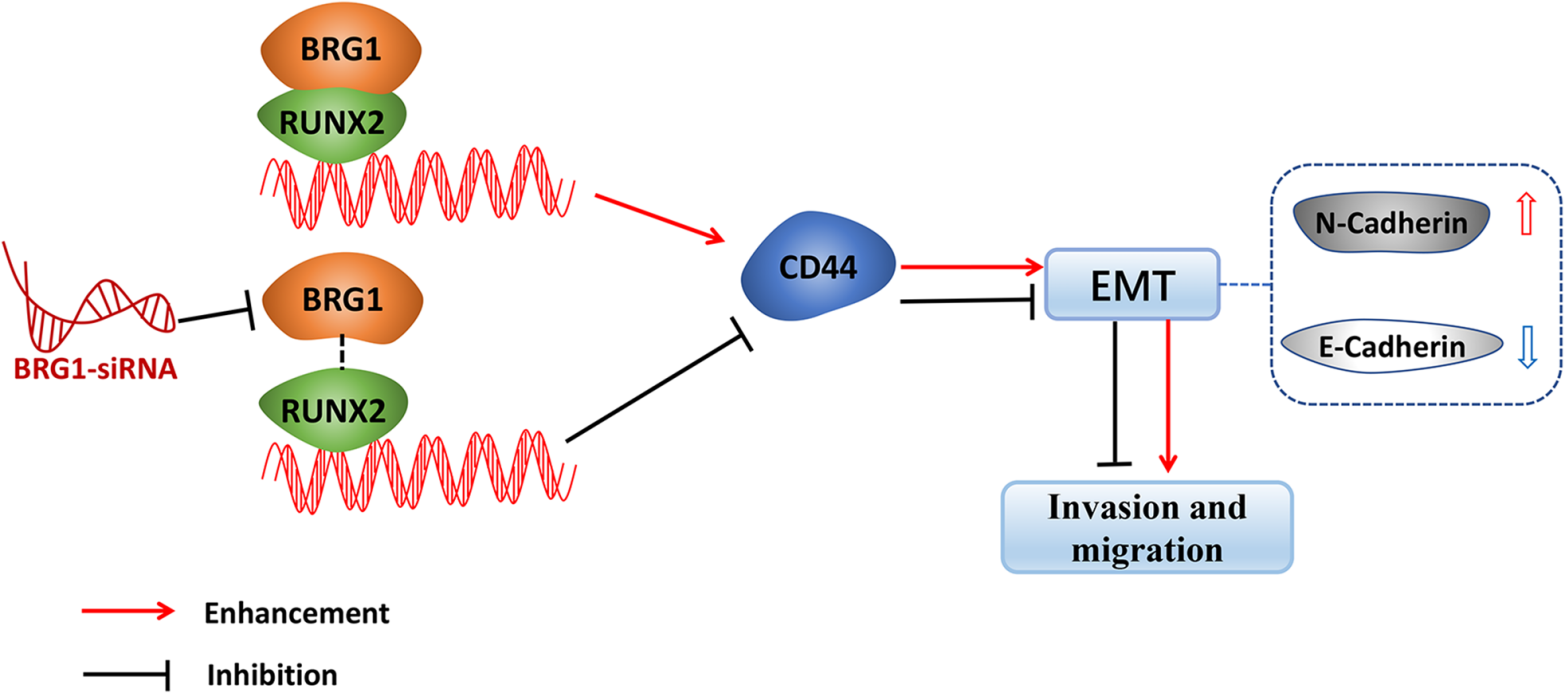
Model of the interaction and relationship among RUNX2, BRG1, CD44, and CRC. RUNX2 synergizes with BRG1 for promoting the CD44 expression as well as for enhancing the EMT process and the invasion and migration of CRC cells. Knockdown of BRG1 with specific si-RNA could markedly inhibit the RUNX2-mediated upregulation of the CD44 expression, suggesting that RUNX2 promotes the CD44 expression and EMT depending on the interaction between RUNX2 and BRG1 in CRC cells

RUNX2 acts as a transcription factor and regulates the biological activities of tumors depending on their interacting proteins [47]. BRG1 is the central catalytic subunit of several chromatin-modifying enzyme complexes that use the energy generated by ATP hydrolysis in order to destroy the chromatin structure of the target promoter [48]. It is extremely important to balance the chromosomal remodeling activity for the cell growth response to the surrounding environmental factors so as to prevent the malignant transformation of normal cells [49]. The role of BRG1 may differ in different tissues, albeit the structural or functional changes of BRG1 can influence the increase or decrease of the binding of a transcription factor to chromatin [25, 50–52]. Previous studies have shown that BRG1 is required to enable the expression of CD44 in tumor cells as well as to provide a mechanism for the activation of CD44 expression depending on the chromatin remodeling activity mediated by BRG1 [24]. Consistent with the past findings, our results revealed that BRG1 can regulate the CD44 expression as well as the sphere-forming ability of CRC cells. Moreover, we first found that RUNX2 can bind to BRG1 and that the knockdown of BRG1 could markedly inhibit the RUNX2-mediated upregulation of the CD44 expression, suggesting that RUNX2 promotes the CD44 expression and EMT depending on the interaction between RUNX2 and BRG1 in CRC cells. In addition, the results of the CHIP-qPCR assays revealed that the knockdown of BRG1 could block the binding of RUNX2 to the CD44 promoter, which in turn indicates that BRG1 contributes to the recruitment of RUNX2 to the CD44 promoter. Some studies also have shown that BRG1 is involved in the regulation of CD44 demethylation [53], however, whether RUNX2-BRG1 complex regulates the CD44 expression and EMT is related to the BRG1-mediated gene demethylation remains to be further studied. Thus, along with the finding that RUNX2 can promote the EMT through the CD44 pathway, our cumulative findings suggest that RUNX2 synergizes with BRG1 for promoting the CD44 expression as well as for enhancing the EMT process. The present report is the first demonstration of the interaction of the transcription factor RUNX2 with BRG1 for the regulation of the CD44 expression toward promoting the invasion and migration in CRC cells (Fig. 6).

Meanwhile, we also analyzed the protein and mRNA expressions of RUNX2, BRG1, and CD44 in the CRC tissues and found that their protein and mRNA levels were more elevated in the CRC tissues than in the adjacent cancer tissues. Their correlational analysis at the mRNA expression level suggested the existence of a significant positive correlation among RUNX2, BRG1, and CD44 as well as that between BRG1 and CD44. The cBio Cancer Genomics Portal (https://cbioportal.org) is an open platform for the interactive exploration of multidimensional cancer genomics data sets [40]. We employed the corresponding RNA-Seq data from cBioPortal for Cancer Genomics database to analyze the correlation among the mRNA expressions of RUNX2, BRG1, and CD44 supporting the above observations. Of course, our research still has some limitations. First of all, in the study of clinical samples, we only included cases from 2010 to 2013, and there is no relevant analysis of survival and prognosis. In addition, this is just an in vitro mechanism study, which is still lack of in vivo validation, and we will further improve the pertinent research content in subsequent studies.

In general, we found that RUNX2 can promote the formation of stem cells and the invasion and migration in CRC cells. Further research suggested that RUNX2 could interact with BRG1 by forming a compact complex for the regulation of the transcription and expression of CD44. This research provides a new clue for regarding the mechanism of RUNX2 promoting the invasion and metastasis of CRC, thereby providing a new research target for the prevention and treatment of CRC recurrence and metastasis.

The tumorigenesis and progression of CRC involves multiple genes. With the increase in the oncogenic functions and the loss of tumor suppressor gene functions, the cells demonstrated uncontrolled inhibition of proliferation, differentiation, and apoptosis [14, 15]. Therefore, we committed to identifying highly specific CRC-related genes in clinical practice to provide the basis for the diagnosis, targeted treatment, and prognosis risk assessment of CRC through the combined detection of multiple genes.

## Conclusion

The present study revealed that RUNX2 is critical for the sustenance of the stem cell-like properties of CRC cells as well as for the promotion of CD44-induced EMT in CRC cells. In addition, RUNX2 can interact with BRG1 by forming a compact complex to regulate the transcription and expression of CD44, which has possible involvement in the invasion and migration of CRC cells. We believe that our present findings would add to the current understanding of RUNX2-mediated carcinogenesis in CRC as well as provide a novel insight to multi-gene-combined diagnosis and treatment of CRC.

## Supplementary information

**Additional file 1:** **Table S1.** Key resources table. **Table S2.** RT-qPCR primers.

## Data Availability

The datasets generated and/or analyzed during the current study are available from the corresponding author on reasonable request.

## Abbreviations

CSC: Cancer stem cell
EMT: Epithelial–mesenchymal transition
CRC: Colorectal cancer
RUNX2: Runt-related transcription factor-2
RT-qPCR: Real-time quantitative PCR
siRNA: Small-interfering RNA
PBS: Phosphate-buffered saline
co-IP: Coimmunoprecipitation
CHIP: Chromatin immunoprecipitation
IHC: Immunohistochemistry

## References

[1] Batlle E, Clevers H (2017). Cancer stem cells revisited. Nat Med.

[2] Nandy SB, Lakshmanaswamy R (2017). Cancer stem cells and metastasis. Prog Mol Biol Transl Sci.

[3] Toh TB, Lim JJ, Chow EK (2017). Epigenetics in cancer stem cells. Mol Cancer.

[4] Jing F (2015). Colon cancer stem cell markers CD44 and CD133 in patients with colorectal cancer and synchronous hepatic metastases. Int J Oncol.

[5] Tsunekuni K (2019). CD44/CD133-positive colorectal cancer stem cells are sensitive to trifluridine exposure. Sci Rep.

[6] Wang L (2018). The role of CD44 and cancer stem cells. Methods Mol Biol.

[7] Ibrahim HM (2019). Prognostic value of cyclin D1 and CD44 expression in gastric adenocarcinoma. J Gastrointest Cancer.

[8] Chaffer CL (2016). EMT, cell plasticity and metastasis. Cancer Metastasis Rev.

[9] Gloushankova NA, Zhitnyak IY, Rubtsova SN (2018). Role of epithelial-mesenchymal transition in tumor progression. Biochemistry (Mosc).

[10] Huang L, Wu RL, Xu AM (2015). Epithelial-mesenchymal transition in gastric cancer. Am J Transl Res.

[11] Vu T, Datta PK (2017). Regulation of EMT in colorectal cancer: a culprit in metastasis. Cancers (Basel).

[12] Loh CY (2019). The E-Cadherin and N-Cadherin Switch in epithelial-to-mesenchymal transition: signaling, therapeutic implications, and challenges. Cells.

[13] Leng Z (2018). Lgr5+CD44+EpCAM+ strictly defines cancer stem cells in human colorectal cancer. Cell Physiol Biochem.

[14] Long AG, Lundsmith ET, Hamilton KE (2017). Inflammation and colorectal cancer. Curr Colorectal Cancer Rep.

[15] Javan B, Shahbazi M (2018). Constructing a novel hypoxia-inducible bidirectional shRNA expression vector for simultaneous gene silencing in colorectal cancer gene therapy. Cancer Biother Radiopharm.

[16] Sase T (2012). Runt-related transcription factor 2 in human colon carcinoma: a potent prognostic factor associated with estrogen receptor. Int J Cancer.

[17] Komori T (2019). Regulation of proliferation, differentiation and functions of osteoblasts by RUNX2. Int J Mol Sci.

[18] Ozaki T (2018). Impact of RUNX2 on drug-resistant human pancreatic cancer cells with p53 mutations. BMC Cancer.

[19] Wang Q (2016). RUNX2 promotes hepatocellular carcinoma cell migration and invasion by upregulating MMP9 expression. Oncol Rep.

[20] Valenti MT (2016). Runx2 expression: A mesenchymal stem marker for cancer. Oncol Lett.

[21] Mevel R (2019). RUNX transcription factors: orchestrators of development. Development.

[22] Knutson TP (2017). Posttranslationally modified progesterone receptors direct ligand-specific expression of breast cancer stem cell-associated gene programs. J Hematol Oncol.

[23] Herreno AM (2019). Role of RUNX2 transcription factor in epithelial mesenchymal transition in non-small cell lung cancer lung cancer: Epigenetic control of the RUNX2 P1 promoter. Tumour Biol.

[24] Strobeck MW (2001). The BRG-1 subunit of the SWI/SNF complex regulates CD44 expression. J Biol Chem.

[25] Kuo KT (2006). Downregulation of BRG-1 repressed expression of CD44s in cervical neuroendocrine carcinoma and adenocarcinoma. Mod Pathol.

[26] Xi Q (2008). Genome-wide impact of the BRG1 SWI/SNF chromatin remodeler on the transforming growth factor beta transcriptional program. J Biol Chem.

[27] Sun J (2019). TRIM29 facilitates the epithelial-to-mesenchymal transition and the progression of colorectal cancer via the activation of the Wnt/beta-catenin signaling pathway. J Exp Clin Cancer Res.

[28] Peng F (2017). H19/let-7/LIN28 reciprocal negative regulatory circuit promotes breast cancer stem cell maintenance. Cell Death Dis.

[29] Wasserman WW, Sandelin A (2004). Applied bioinformatics for the identification of regulatory elements. Nat Rev Genet.

[30] Wang G (2017). Loss of BRG1 induces CRC cell senescence by regulating p53/p21 pathway. Cell Death Dis.

[31] Wu DM (2019). The PAX6-ZEB2 axis promotes metastasis and cisplatin resistance in non-small cell lung cancer through PI3K/AKT signaling. Cell Death Dis.

[32] Yamada D (2018). RUNX2 promotes malignant progression in glioma. Neurochem Res.

[33] Lu H (2018). RUNX2 plays an oncogenic role in esophageal carcinoma by activating the PI3K/AKT and ERK signaling pathways. Cell Physiol Biochem.

[34] Jurkovicova D (2016). Evaluation of expression profiles of microRNAs and two target genes, FOXO3a and RUNX2, effectively supports diagnostics and therapy predictions in breast cancer. Neoplasma.

[35] Yeung KT, Yang J (2017). Epithelial-mesenchymal transition in tumor metastasis. Mol Oncol.

[36] Cho SH (2012). CD44 enhances the epithelial-mesenchymal transition in association with colon cancer invasion. Int J Oncol.

[37] Muthuswami R (2019). BRG1 is a prognostic indicator and a potential therapeutic target for prostate cancer. J Cell Physiol.

[38] Raab JR (2017). Co-regulation of transcription by BRG1 and BRM, two mutually exclusive SWI/SNF ATPase subunits. Epigenetics Chromatin.

[39] Lan J (2017). BRG1 promotes VEGF-A expression and angiogenesis in human colorectal cancer cells. Exp Cell Res.

[40] Cerami E (2012). The cBio cancer genomics portal: an open platform for exploring multidimensional cancer genomics data. Cancer Discov.

[41] Gorgen A (2018). The new era of transplant oncology: liver transplantation for nonresectable colorectal cancer liver metastases. Can J Gastroenterol Hepatol.

[42] Villanueva F (2019). The cancer-related transcription factor RUNX2 modulates expression and secretion of the matricellular protein osteopontin in osteosarcoma cells to promote adhesion to endothelial pulmonary cells and lung metastasis. J Cell Physiol.

[43] Wang XX (2018). Serum miR-4530 sensitizes breast cancer to neoadjuvant chemotherapy by suppressing RUNX2. Cancer Manag Res.

[44] Qin L (2016). MicroRNA-455 regulates migration and invasion of human hepatocellular carcinoma by targeting RUNX2. Oncol Rep.

[45] Zhu X, Niu X, Ge C (2019). Inhibition of LINC00994 represses malignant behaviors of pancreatic cancer cells: interacting with miR-765-3p/RUNX2 axis. Cancer Biol Ther.

[46] Wang ZQ (2013). Inhibition of RUNX2 transcriptional activity blocks the proliferation, migration and invasion of epithelial ovarian carcinoma cells. PLoS ONE.

[47] Yang S (2015). Subnuclear domain proteins in cancer cells support the functions of RUNX2 in the DNA damage response. J Cell Sci.

[48] Barutcu AR (2016). SMARCA4 regulates gene expression and higher-order chromatin structure in proliferating mammary epithelial cells. Genome Res.

[49] Hota SK, Bruneau BG (2016). ATP-dependent chromatin remodeling during mammalian development. Development.

[50] Laurette P (2015). Transcription factor MITF and remodeller BRG1 define chromatin organisation at regulatory elements in melanoma cells. Elife.

[51] Sethy R (2018). Regulation of ATM and ATR by SMARCAL1 and BRG1. Biochim Biophys Acta Gene Regul Mech.

[52] Kim KH (2015). SWI/SNF-mutant cancers depend on catalytic and non-catalytic activity of EZH2. Nat Med.

[53] Banine F (2005). SWI/SNF chromatin-remodeling factors induce changes in DNA methylation to promote transcriptional activation. Cancer Res.

