# Supplementary material for: RUNX2 interacts with BRG1 to target CD44 for promoting invasion and migration of colorectal cancer cells
Source: Cancer Cell Int. 2020 Oct 15;20:505. doi: 10.1186/s12935-020-01544-w (PMC7559818; doi:10.1186/s12935-020-01544-w)
Supplement: Supplementary file 1 — Additional file 1: Table S1. Key resources table. Table S2. RT-qPCR primers. [file 12935_2020_1544_MOESM1_ESM.docx]

**Supplementary table 1. Key resources table**

| **Reagent type  (species) or  resource** | **Designation** | **Source or reference** | **Identifiers** | **Additional information** |
| --- | --- | --- | --- | --- |
| Antibody | Rabbit monoclonal anti-RUNX2 | Cell signaling Technology | Cat. # 12556S | (1:1000) for western blot (WB)  (1:50) for immunoprecipitation (IP) |
| Antibody | Rabbit (DA1E) mAb IgG XP® Isotype Control | Cell signaling Technology | Cat. # 3900S | Dilute to the same concentration as the specific IP antibody |
| Antibody | normal mouse IgG | Santa Cruz Biotechnology | Cat. # sc-2025 | Dilute to the same concentration as the specific IP antibody |
| Antibody | Mouse monoclonal anti – RUNX2 | Abcam | Cat. # ab76956 | (1:100) for immunofluorescence (IF).  (1:200) for Immunohistochemistry (IHC) |
| Antibody | Rabbit Polyclonal anti- SMARCA4/BRG1 | Proteintech | Cat. # 21634-1-AP | (1:1000) for WB  (1μg/mg protein) for IP  (1:200) for IF  (1:200) for IHC |
| Antibody | Mouse monoclonal anti-CD44 | R&D Systems | Cat. # BBA10 | 2 µg/mL for WB  10 µg/mL for IHC |
| Antibody | Rabbit Polyclonal anti- E-cadherin | Proteintech | Cat. # 20874-1-AP | (1:2000) for WB |
| Antibody | Rabbit Polyclonal anti- N-cadherin | Proteintech | Cat. # 22018-1-AP | (1:2000) for WB |
| Antibody | Goat anti-Mouse IgG (H+L) Cross-Adsorbed Secondary Antibody, Alexa Fluor 594 | ThermoFisher SCIENTIFIC | Cat. # A-11005 | (1:200) for IF |
| Antibody | Goat anti-Rabbit IgG (H+L) Highly Cross-Adsorbed Secondary Antibody, Alexa Fluor 488 | ThermoFisher SCIENTIFIC | Cat. # A-11034 | (1:200) for IF |
| HRP-linked secondary antiboay | Rabbit monoclonal antiboay IgG | Cell signaling Technology | Cat. # 7074S | (1:5000) for WB  (1:5000) for IHC |
| HRP-linked secondary antiboay | Mouse monoclonal antiboay IgG | Cell signaling Technology | Cat. # 7076S | (1:5000) for WB  (1:5000) for IHC |
| Antibody | Rabbit monoclonal anti-β-Actin | Cell signaling Technology | Cat. # 4970S | (1:1000) for WB |
| Antibody | Rabbit Polyclonal  anti-GFP Tag | Proteintech | Cat. # 50430-2-AP | (1:1000) for WB  (1μg/mg protein) for IP |
| Antibody | Myc-Tag (9B11) Mouse monoclonal antiboay | Cell signaling Technology | Cat. # 2276S | (1:1000) for WB  (1:100) for IP |

**Supplementary table 2. RT-qPCR primers**

| Primers | Sequence(5’to3’) |
| --- | --- |
| RUNX2 - Forward | ACTACCAGCCACCGAGACCA |
| RUNX2 - Reverse | ACTGCTTGCAGCCTTAAATGACTCT |
| BRG1 - Forward | CAGATCCGTCACAGGCAAAAT |
| BRG1 - Reverse | TCTCGATCCGCTCGTTCTCTT |
| CD44 - Forward | CAGACCTGCCCAATGCCTTTGATGGACC |
| CD44 - Reverse | CAAAGCCAAGGCCAAGAGGGATGCC |
| GAPDH - Forward | CCTCGTCTCATAGACAAGATGGT |
| GAPDH - Reverse | GGGTAGAGTCATACTGGAACATG |
